# Historical Trends in Sweet Corn Plant Density Tolerance Using Era Hybrids (1930–2010s)

**DOI:** 10.3389/fpls.2021.707852

**Published:** 2021-09-22

**Authors:** Daljeet S. Dhaliwal, Elizabeth A. Ainsworth, Martin M. Williams

**Affiliations:** ^1^Department of Crop Sciences, University of Illinois at Urbana Champaign, Urbana, IL, United States; ^2^Global Change and Photosynthesis Research Unit, United States Department of Agriculture – The Agricultural Research Service (USDA-ARS), Urbana, IL, United States

**Keywords:** plant density tolerance, *Zea mays*, yield potential, hybrid era, factor analysis

## Abstract

Over the last six decades, steady improvement in plant density tolerance (PDT) has been one of the largest contributors to genetic yield gain in field corn. While recent research indicates that PDT in modern sweet corn hybrids could be exploited to improve yield, historical changes in PDT in sweet corn are unknown. The objectives of this study were to: (a) quantify the extent to which PDT has changed since introduction of hybrid sweet corn and (b) determine the extent to which changes over time in PDT are associated with plant morpho-physiological and ear traits. An era panel was assembled by recreating 15 *sugary1* sweet corn hybrids that were widely used at one time in the United States, representing hybrids since the 1930s. Era hybrids were evaluated in field experiments in a randomized complete block design with a split-plot arrangement of treatments, including hybrid as the main factor and density as the split-plot factor. Plant density treatments included “Low” plant density (9,900 plants/ha) free of crowding stress or “High” plant density (79,000 plants/ha) with crowding stress. On average, per-area marketable ear mass (Mt/ha) increased at a rate of 0.8 Mt/ha/decade at High densities, whereas per-plant yield (i.e., kg/plant) remained unchanged over time regardless of the density level. Crate yield, a fresh market metric, improved for modern hybrids. However, processing sweet corn yield metrics like fresh kernel mass and recovery (amount of kernel mass contributing to the fresh ear mass) showed modest or no improvement over time, respectively. Modern sweet corn hybrids tend to have fewer tillers and lower fresh shoot biomass, potentially allowing the use of higher plant density; however, plant architecture alone does not accurately predict PDT of individual hybrids.

## Introduction

Grain yield gains in field corn over the past six decades have attributed to genetic gains and improved management practices (Tollenaar et al., 1994; Duvick, 2001). An estimated 50–70% of yield gains are due to improved genetics, with the remaining attributed to superior management practices (Duvick, 2001). Genetic gains are associated with increased plant density tolerance (PDT, also known as crowding stress tolerance) in modern field corn hybrids (Tollenaar and Wu, 1999), as evidenced by increases in plant density at a rate of ∼700 plants/ha/year from 1987 to 2016 (Assefa et al., 2018).

Previous research has shown variability for PDT among widely used sweet corn hybrids (Williams, 2015). Sweet corn hybrids with improved PDT, when planted at their optimum plant densities, outperform hybrids with poor PDT (Williams, 2012). Recent research has shown that both vegetable processors and sweet corn growers benefit from using higher (i.e., economic optimum) plant densities for PDT hybrids without changing other management practices (Dhaliwal and Williams, 2019). While there is evidence that improved PDT in sweet corn could be exploited in ways to increase profitability for the sweet corn industry, the extent to which PDT has changed since introduction of hybrid sweet corn remains an open empirical question.

Numerous studies have reported on plant morpho-physiological traits associated with improved PDT in field corn (Tetio-Kagho and Gardner, 1988; Sangoi et al., 2002; Duvick, 2005). More recent research evaluated 48 phenotypic traits from five categories (photosynthetic capability, plant architecture, growth responses, source–sink relationship, and general stress tolerance) in relation to PDT in field corn (Mansfield and Mumm, 2014). Williams (2016) reported two categories of traits, namely, photosynthetic capacity and source–sink relationships associated with PDT in modern *shrunken-2* processing sweet corn hybrids. However, traits involved with changes in PDT over time in sweet corn remain to be explored. This knowledge gap is significant, because not only are sweet corn hybrids unique, but the yield metric of field corn (i.e., grain) does not apply to critical yield metrics of fresh market or processing sweet corn.

Using an era panel of *sugary1* (*su1*) sweet corn hybrids, the objectives of this study were to: (a) quantify the extent to which PDT has changed since introduction of hybrid sweet corn and (b) determine the extent to which changes over time in PDT are associated with plant morpho-physiological and ear traits.

## Materials and Methods

### Germplasm

An era panel of 15 *su1* sweet corn hybrids was created (Table 1). While some older hybrids are commercially available today (e.g., Golden Cross Bantam), many were not. Older, important hybrids no longer commercially available were recreated in-kind exclusively for this experiment by participating seed or processing companies. The entries represent some of the most widely used hybrids, by acreage, during their zenith since introduction of hybrid sweet corn in the 1930s.

**TABLE 1:** Basic information about the *sugary1* sweet corn hybrids evaluated for plant density tolerance in field trials at Urbana, IL, in 2018–2020.

Hybrid	Year of release	Source
Golden Cross Bantam	1934	Charter Seed Company
IowaChief	1951	Charter Seed Company
NK199	1954	Charter Seed Company
Jubilee	1959	Syngenta
Silver Queen	1960	Syngenta
Merit	1961	Seminis
StylePak	1975	Harris Moran Seed Company
DMC2038	1984	DelMonte
Chase	1988	Seminis
Eliminator	1993	Crookham Company
Bonus	1995	Syngenta
Golden Beauty	1995	Charter Seed Company
GH6462	2004	Syngenta
SC1263	2010	Seminis
GH9394	2014	Syngenta

### Site Description

The study was conducted near Urbana, IL at the University of Illinois Vegetable Crop Research Farm (40°04′36.0″N 88°14′35.7″W) from 2018 to 2020. The predominant soil type is a Flanagan silt loam (fine, smectitic, mesic Aquic Argiudolls) with 5.8% organic matter. The previous crop for all years was soybean [*Glycine max* (L.)] in a sweet corn–soybean rotation. Growing season conditions for all three years are provided in Supplementary Figure 1.

### Experimental Design

The experiment was a randomized complete block with four replicates and treatments assigned in a split-plot arrangement of treatments. The main plot factor was the hybrid line, and subplots were assigned plant density factor (9,900 and 79,000 plants/ha). Hereafter, the two levels of plant density will be simple referred to as “Low” and “High” plant density. Low plant density represents growing conditions free of crowding stress. High plant density was chosen to induce crowding stress based on previous research (Williams, 2015). The dimensions of main plots were 9.1 m by 6.1 m, and each four-row subplot (76 cm row spacings) measured 9.1 m by 3 m. The study was planted on a different field each year on May 15, June 1, and June 1 in 2018, 2019, and 2020, respectively. The seed bed was prepared by a single pass of a field cultivator prior to planting. The study was overseeded at planting to improve seedling recruitment, and subplots were thinned to the desired levels of plant density at the two-leaf stage. Tefluthrin {(2,3,5,6-tetrafluoro4-methylphenyl) methyl (*1R,3R*)-rel-3-[(*1Z*)-2-chloro-3,3,3-trifluoro-1-propenyl]-2,2-dimethylcyclopropanecarboxylate} was applied in a t-band at planting to control corn rootworms (*Diabrotica* spp.) A pre-emergence treatment of s-metolachlor {2-chloro-N-(2-ethyl-6-methylphenyl)-N-[(1S)-2-methoxy1-methylethyl acetamide} plus atrazine (2-chloro-4-ethylamino-6-isopropylamino-1,3,5-triazine) was applied after planting. The study was kept weed-free by hand weeding and a post-emergence treatment of 1 kg/ha a.i. atrazine (2-chloro-4-ethylamino-6-isopropylamino-1,3,5-triazine) in 2019 and 2020. The plots were irrigated using a linear irrigation system to avoid water deficit stress during periods of abnormally low rainfall.

### Data Collection

Mid-tassel (VT) and mid-silk (R1) dates were recorded for each subplot. Beginning at tassel emergence, the total number of plants with fully opened tassel branches was counted until at least 50% of the total plants in the center two rows of a subplot had fully opened tassel branches. Similarly, mid-silking date was recorded by counting the total number of plants with visible silks on primary ears until at least one-half of the total plants in center rows reached R1. The difference between mid-anthesis and mid-silking dates was used to determine the anthesis–silking interval (ASI). Cumulative growing degree days (GDD) using a base temperature 10°C and daily air temperature data were recorded from a weather station within 1 km of the experiment sites. Cumulative GDDs from planting to silking and GDD accumulation over the ASI were calculated. Growth degree days were calculated using the below equation:

$$G\,D\,D=\left[\left(T_{m\,a\,x}-T_{m\,i\,n}\right)/2\right]-T_{b\,a\,s\,e}$$

where *T*_*max*_ and *T*_*min*_ are the daily maximum and minimum air temperature, respectively, and *T*_*base*_ is the base temperature (here 10°C).

#### Plant Morphological Measurements

All plant morphological traits were measured at silking stage on two randomly selected plants from the center two rows of each subplot. Plant flag leaf height and primary ear height were measured from the soil surface. Leaf angle was measured on the 10th leaf of randomly selected plants using a clinometer smartphone application. Leaf angle was measured as the angle of leaf relative to the stalk; thereby, more upright leaf would have smaller angle. Leaf number and tillers per plant were recorded. Leaf area index (LAI) was estimated in full sun within 2 h of solar noon with a linear ceptometer (AccuPAR Linear Ceptometer; Decagon Devices, Pullman, WA, United States) for the center two rows of each subplot.

#### Physiological Data

Leaf gas exchange was measured at midday at silking on the leaf subtending the primary ear using four portable gas exchange systems (LI-6800, LICOR, Lincoln, NE, United States) with the leaf cuvette set to ambient conditions measured at the leaf subtending the ear: (CO_2_) (410 mmol mol^–1^), temperature (28.2–32.4°C), light level (750–1,500 μmol m^–2^ s^–1^), vapor pressure deficit (1.1–1.8 kPa). The flow rate was set to 500 mmol s^–1^. Within each year of measurement, all gas exchange systems were set to the same temperature and light levels to ensure consistency between measurements within a growing season. Leaf photosynthesis (*A*) and stomatal conductance to water vapor (g_*s*_) were calculated using the equations of von Caemmerer and Farquhar (1981). Instantaneous water use efficiency was calculated as *A*/g_*s*_.

#### Harvest Data

Plots were hand harvested at the milk stage (R3) of development, which was 18–21 days after mid-silk. Six meters of the center two rows for each subplot was harvested, and stand counts were recorded for the 6 m harvest length. Green ears with diameter >4.5 cm were considered “marketable” ears; smaller ears were considered “non-marketable.” Marketable and non-marketable ear mass and number were recorded for each subplot. Marketable ear mass per plant was calculated as the total marketable mass divided by stand count over the harvest length for each subplot. Similarly, marketable ear number was calculated using marketable ear number and stand counts over the harvest length for each subplot. Marketable ear number was used to estimate crate yield (crates/ha)—a commonly used metric in the fresh-market industry, with each crate containing 48 ears. A subsample of 10 randomly selected marketable ears was measured for ear traits described below. Subsampled green ears were husked with a husking bed (A&K Development, Eugene, OR, United States). Husked ear mass, ear length, and filled ear length were recorded. Fresh kernels were cut from the cob using an industry-grade hand-fed corn cutter (A&K Development, Eugene, OR, United States). Cob mass was recorded. Kernel mass was calculated as the difference between husked ear mass and cob mass. Recovery was calculated as the percentage of green ear mass constituted by kernel mass. A subsample of kernel mass (∼100 g) was used to determine kernel moisture content gravimetrically at 55°C until dry. Kernel moisture was adjusted to 76%.

### Statistical Analyses

#### Plant Morpho-Physiological and Yield and Ear Traits

All response variables were analyzed with an analysis of variance (ANOVA) model using the mixed procedure in SAS (version 9.4; Sas Institute, 2020). The Shapiro–Wilk test of normality and Brown–Forsythe test for homogeneity of variance were performed on ANOVA residuals to test model assumptions. As needed, the Box-Cox procedure (Box and Cox, 1964) was used to transform response variables to satisfy model assumptions. Plant density, hybrid, and their interactions were considered fixed effects. Year and replicates nested within year were treated as random effects. Mean comparisons for significant treatment effects were performed using Tukey’s mean separation test (α = 0.05).

#### Regression Analysis and Comparison of Slope Estimates

Simple linear regression models were constructed to quantify changes over time in response variables with significant plant density by hybrid interaction effects. Data were analyzed separately for each year (2018–2020).

$${\text{Y}}_{i\,j}=\beta_{0}+\beta_{1}\,\text{Y}\,O\,R_{i}+\beta_{2}\,{\text{D}}_{j}+\beta_{3}\,\text{Y}\,O\,R_{i}\,{\text{D}}_{j}+\varepsilon_{i\,j}$$

**Y**_**i***j*_ is the response variable for ***i*^*th*^** year of release and ***j*^*th*^** plant density,

**Y***OR*_**i**_ is the ***i***^*th*^ year of release for hybrid,

**D_j_** is the ***j***^*th*^ plant density level, where $\left\{ \begin{array}{l} j=0,\;if\;plant\;density\;is\;9,900\;plants/ha\\ j=1,\;if\;plant\;density\;is\;79,000\;plants/ha \end{array}\right.$

**Y***OR*_**i**_**D**_**j**_ is the interaction between ***i*^*th*^** year of release for hybrid and ***j*^*th*^** plant density

ε_**i***j*_ is the random error term associated with response variable **Y**_**i***j*_, and ε_**i***j*_∼**N**[**0**,σ^2^].

A significant interaction term indicates slope estimates for Low and High plant density levels were different at α = 0.05.

#### Factor Analysis and Factor Regression

A correlation matrix of plant morpho-physiological and ear traits was used for exploratory factor analysis to reduce dimensionality of data. Low and High plant density data were analyzed separately using *stats* package in R (R Core Team, 2020) with varimax rotation. Factors with eigenvalues >1 were retained, and the orthogonal factor loadings for each latent factor were interpreted similar to correlation coefficients. Factor scores matrix was obtained by multiplying factor loadings matrix and standardized plant morpho-physiological and ear trait variables used for factor analysis.

Partial correlation coefficients were obtained for factor scores and per-area marketable ear mass (Mt/ha), separately for Low and High plant densities. Factor scores for the latent variables and year of release for hybrid were used as independent variables to predict per-area marketable ear mass (Mt/ha) using separate linear regression models for Low and High plant densities.

## Results

### Yield and Ear Traits

Plant density and hybrid had an interactive effect on yield traits including per-area marketable ear mass (Mt/ha), crate yield (crates/ha), per-plant marketable ear mass (kg/plant), and number of marketable ears per plant (Table 2A). High plant densities reported higher per-area marketable ear mass, while Low densities showed higher per-plant marketable ear mass. All ear traits except recovery were greater under Low densities.

**TABLE 2:** Significance of fixed effects and interactions for crop response variables as a function of plant density and sweet corn hybrid for **(A)** Yield and Ear traits, **(B)** Growth and Development traits, and **(C)** Physiological traits at Urbana, IL in 2018–2020.

(A) Yield (area-wise and per plant) and ear traits									
		Area yield			Yield per plant		Ear traits		
Main effects		Per-area marketable ear mass	Kernel mass	Crate yield	Per-plant marketable ear mass	Marketable ears	Ear length	Filled ear length	Recovery
		Mt/ha	Mt/ha	Crates/ha	kg/plant	no./plant	cm	%	%
Plant density (D)		^**^	^**^	^**^	^**^	^**^	^**^	^**^	^**^
	Low	7.6	3.2	413	0.73	2.0	19.5	18.7	39.4
	High	14.4	6.9	1,064	0.18	0.7	18.3	16.7	41.1
Hybrid (H)		^**^	^**^	^**^	^**^	^**^	^**^	^**^	^**^
Interaction									
	DxH	^**^	NS	^**^	^**^	^**^	^*^	NS	NS

**(B) Growth and development traits**										

**Main effects**		**Flag leaf height**	**Primary ear height**	**Tiller number**	**Leaf number**	**Leaf angle**	**Leaf Area Index**	**Shoot biomass**	**Days to silking**	**Anthesis-silking interval**
		cm	cm	no./plant	no./plant	degrees	–	g/plant	GDD	GDD

Plant density (D)		^**^	^**^	^**^	NS	^**^	^**^	^**^	^**^	NS
	Low	152.9	54.1	2.2	14.9	41.8	2.12	1.17	1,305	104
	High	165.9	61.6	0.8	14.8	38.3	4.14	0.37	1,324	104
Hybrid (H)		^**^	^**^	^**^	^**^	^**^	^**^	^**^	^**^	^**^
Interaction										
	DxH	NS	NS	^**^	NS	NS	^**^	^**^	NS	NS

**(C) Physiological traits (leaf gas exchange measurements)**				

**Main effects**			**Photosynthetic CO_2_ assimilation**			**Stomatal conductance**		**Instantaneous water use efficiency**		

			μmol m^–2^ s^–1^			mol m^–2^ s^–1^		–		
Plant density (D)			^**^			^**^		^**^		
		Low	40.0			0.386		116.4		
		High	36.1			0.320		124.7		
Hybrid (H)			NS			NS		NS		
Interaction										
		DxH	^**^			^^*^^		NS		

*All crop response variables were recorded for 15 different sweet corn hybrids (H) at two levels of plant density (D), namely, Low (9,900 plants/ha) and High (79,000 plants/ha).*

^*^ and ^**^ denote significant effects at *p* < 0.05 and *p* < 0.01, respectively. NS stands for a non-significant effect.

### Growth and Development Traits

Plant density influenced most growth and development traits (Table 2B). High plant density favored taller plants with higher position of the flag leaf and height of the primary ear from the soil surface. Plants in the High plant density treatment had fewer tillers per plant, lower fresh shoot biomass, but greater LAI compared to plants in the Low plant density treatment. Only a few variables (i.e., tiller number, LAI, and fresh shoot biomass) were influenced by an interactive effect of plant density and hybrid (Table 2B).

### Physiological Traits

Plant density, not hybrid, had a main effect on all plant physiological variables (Table 2C). Plants at Low density showed higher photosynthetic CO_2_ assimilation and stomatal conductance but lower instantaneous water use efficiency. This could be attributed to the presence of larger canopy gaps in Low density and complete canopy closure in High density. There was also an interactive effect of plant density and hybrid for photosynthetic CO_2_ assimilation, and stomatal conductance.

### Trends in Per-Area and Per-Plant Yields

**FIGURE 1:**
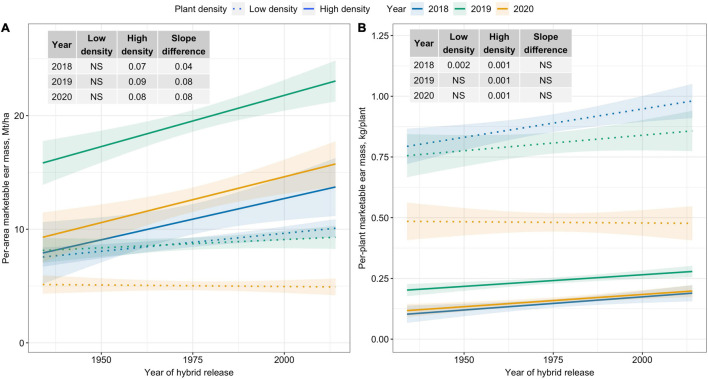
Best fit line for relationship between year of hybrid release and **(A)** per-area marketable ear mass (Mt/ha), and **(B)** per-plant marketable ear mass (kg/plant) for two levels of plant density at Urbana, IL in 2018–2020. Ninety-five percent confidence intervals are shown by the shaded regions around the line of best fit. Slope estimates from linear regression analysis for Low plant density (9,900 plants/ha), High plant density (79,000 plants/ha), and difference between two plant densities are shown. Non-significant slope estimates are denoted by NS (α = 0.05).

Per-area marketable ear mass (Mt/ha) was unchanged over time for Low plant density; however, a significantly increasing trend was observed for High plant density (Figure 1A). Across years, per-area marketable ear mass (Mt/ha) increased by 0.8 Mt/ha for each decade for High density. In contrast, slope estimates for the two densities were similar for per-plant marketable ear mass (kg/plant) (Figure 1B).

### Trends in Yield Metric for Fresh-Market and Processing Industry

**FIGURE 2:**
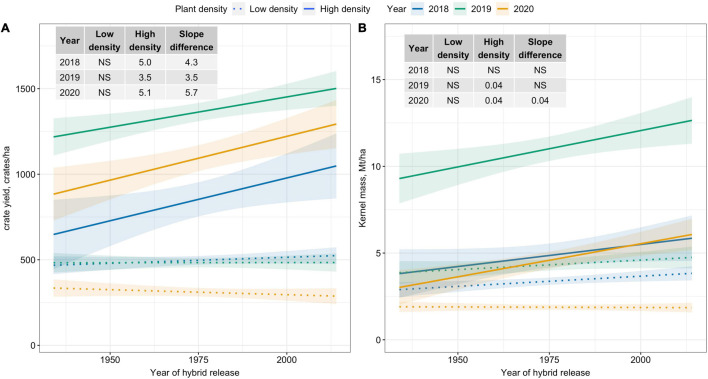
Best fit line for relationship between year of hybrid release and **(A)** crate yield (crates/ha), and **(B)** kernel mass (Mt/ha) for two levels of plant density at Urbana, IL in 2018–2020. Crate yield is the total number of crates, each filled with 48 marketable ears, produced per hectare. Ninety-five percent confidence intervals are shown by the shaded regions around the line of best fit. Slope estimates from linear regression analysis for Low plant density (9,900 plants/ha), High plant density (79,000 plants/ha), and difference between two plant densities are shown. Non-significant slope estimates are denoted by NS (α = 0.05).

**FIGURE 3:**
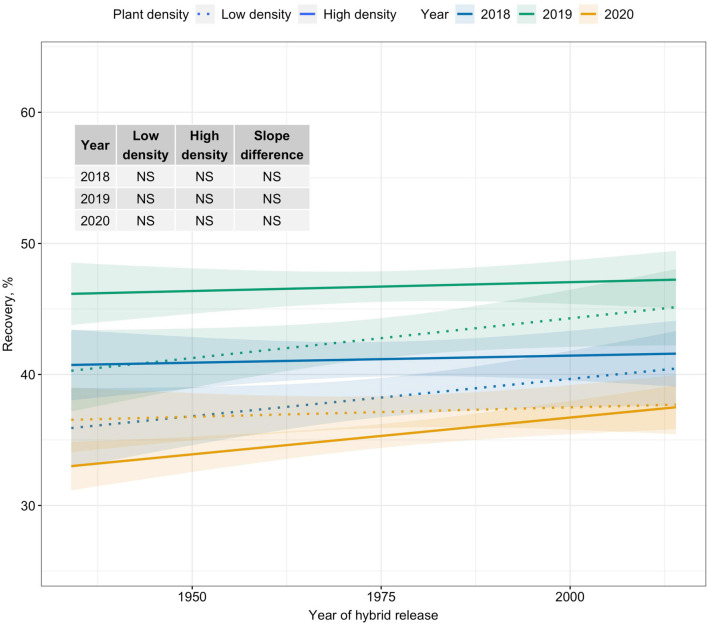
Best fit line for relationship between year of hybrid release and recovery (%) for two levels of plant density at Urbana, IL in 2018–2020. Recovery is the percentage of green ear mass accounted by kernel mass. Ninety-five percent confidence intervals are shown by the shaded regions around the line of best fit. Slope estimates from linear regression analysis for Low plant density (9,900 plants/ha), High plant density (79,000 plants/ha), and difference between two plant densities are shown. Non-significant slope estimates are denoted by NS (α = 0.05).

Crate yield (crates/ha), a yield metric used in the fresh-market industry, increased over time only at High plant density. Crate yield increased by 35–51 crates/ha/decade at High plant density (Figure 2A). Kernel mass (Mt/ha), a yield metric used to evaluate the performance of processing sweet corn, showed slightly increasing trends at High plant density in 2019 and 2020; however, differences in slope estimates between plant densities were inconsistent (Figure 2B). Recovery, an important processor variable showed no trends over the period of 80 years for either density level (Figure 3). Regardless of yield metric used to assess hybrid performance, yield was unchanged over time at Low plant density.

### Trends in Plant Morphological and Ear Traits

**FIGURE 4:**
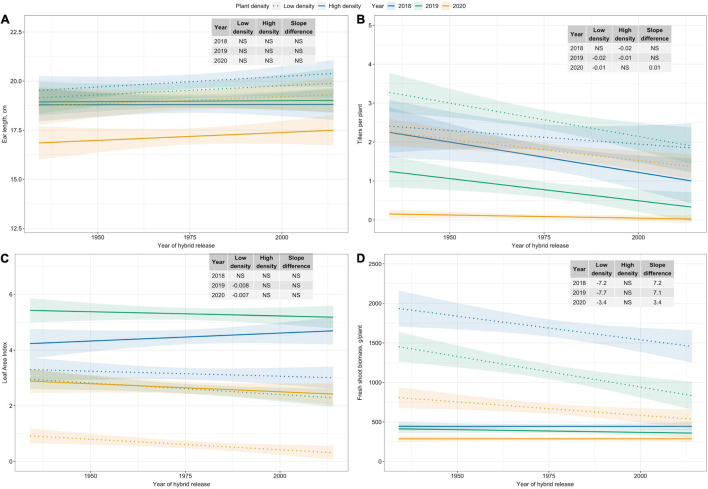
Best fit line for relationship between year of hybrid release and **(A)** ear length (cm), and **(B)** tillers per plant, **(C)** leaf area index, and **(D)** fresh shoot biomass (g/plant) for two levels of plant density at Urbana, IL in 2018–2020. Ninety-five percent confidence intervals are shown by the shaded regions around the line of best fit. Slope estimates from linear regression analysis for Low plant density (9,900 plants/ha), High plant density (79,000 plants/ha), and difference between two plant densities are shown. Non-significant slope estimates are denoted by NS (α = 0.05).

Among plant morphological and ear traits measured, regression analyses for variables with significant plant density–hybrid interactions are illustrated in Figure 4. Ear length has not changed since the 1930s (Figure 4A). However, tillers per plant, LAI, and fresh shoot biomass per plant have generally decreased over time at Low plant density (Figures 4B–D).

### Factor Analysis and Factor Regression

A multivariate approach was used to address the second objective—determine the extent to which changes over time in PDT are associated with plant morpho-physiological and ear traits. Since most of plant morpho-physiological and ear traits were highly correlated and posed issue of multicollinearity in a multiple linear regression model, factor analysis models were used to reduce dimensionality of plant morpho-physiological and ear traits (Table 3). Factors with eigenvalues >1 were retained, resulting in three latent factors for both plant density factor models. Factor models explained 58.6 and 62.0% of the total variability for Low and High plant density, respectively (Table 3). Interestingly, factor loadings of variables on latent factors were similar for both density levels. For instance, physiological variables including transpiration rate, photosynthetic CO_2_ assimilation, stomatal conductance, and instantaneous water use efficiency loaded heavily on Factor 1 for both density levels. Factor 1 can be interpreted as “Physiological traits.” Factor 2 had high loadings for tillers per plant, flag leaf height, LAI, and fresh shoot biomass for both the density levels, and can be inferred as “Plant architecture.” However, Factor 2 for High plant density also had high loadings for ear traits, such as ear length and recovery, in addition to “plant architecture” variables. Factor 3 explained a significant amount of variation for both density levels; however, the factor loadings were from random variables and did not translate into any meaningful latent factor variable.

**TABLE 3:** Exploratory factor analysis results, based on varimax rotation, using the correlation matrix of select ear, growth and development, and leaf gas exchange traits measured at **(A)** Low and **(B)** High plant densities across all sweet corn hybrids at Urbana, IL in 2018–2020.

(A)					
		Low density			
Variable	Units	Factor 1	Factor 2	Factor 3	Communality
Ear length	cm		0.199		0.04
Recovery	%			−**0.416**	0.18
Tillers	No. per plant	–0.208	**0.427**		0.23
Flag leaf ht.	cm	0.127	**0.649**		0.44
LAI	–		**0.793**	–0.232	0.69
Fresh shoot biomass	g/plant	0.111	**0.975**	0.177	1.00
Anthesis-silking interval	GDD	0.153	0.295	0.271	0.18
Photosynthetic CO_2_ assimilation	μmol m^–2^ s^–1^	**0.633**	–0.224	**0.681**	0.92
Stomatal conductance	mol m^–2^ s^–1^	**0.934**		0.340	0.99
Instantaneous water use efficiency	-	−**0.903**	–0.181		0.86
**Variance explained**	**%**	**26.5**	**21.8**	**10.3**	**58.6**

**(B)**					

		**High density**			
**Variable**	**Units**	**Factor 1**	**Factor 2**	**Factor 3**	**Communality**

Ear length	cm		**0.466**		0.23
Recovery	%	–0.276	**0.545**	–0.351	0.50
Tillers	No. per plant		**0.428**		0.19
Flag leaf ht.	cm		**0.763**	0.183	0.62
LAI	–	–0.228	**0.852**		0.79
Fresh shoot biomass	g/plant		**0.730**	0.142	0.55
Anthesis-silking interval	GDD		0.128	**0.460**	0.23
Photosynthetic CO_2_ assimilation	μmol m^–2^ s^–1^	**0.771**	–0.364	**0.437**	0.92
Stomatal conductance	mol m^–2^ s^–1^	**0.978**	–0.116	0.102	0.98
Instantaneous water use efficiency	–	−**0.867**		0.343	0.87
**Variance explained**	**%**	**30.5**	**24.7**	**6.8**	**62.0**

*Factor loadings from variables that were >0.400 in magnitude are in bold.*

Separate multiple linear regression models were conducted for both density levels using factor scores from the factor model and year of release for hybrid as independent variables to predict per-area marketable ear mass (Mt/ha). For both plant densities, increasing scores for Factor 2 resulted in maximum increase in per-area marketable ear mass (Mt/ha) (Table 4). However, the amount of variation in per-area marketable ear mass (Mt/ha) explained by Factor 2 was much higher for the Low plant density (50%) model than the High plant density model (12%) (Table 4). Year of release was positively correlated with per-area marketable ear mass (Mt/ha), but the amount of variation explained was low (≤8%) for both density levels (Table 4).

**TABLE 4:** Regression parameters for per-area marketable ear mass (Mt/ha) as a response of year of hybrid release (YOR) and factor scores for Low and High plant densities across all sweet corn hybrids at Urbana, IL in 2018–2020.

Plant density	Predictor variable	*r* ^*^	Slope estimate	*p*-value	Variance explained (%)
Low	Year of hybrid release	0.44	0.03	<0.001	2.00
	Factor 1	–0.10	−0.17	0.19	–
	Factor 2	0.79	2.04	<0.001	50.0
	Factor 3	–0.30	−0.53	<0.001	4.00
			**Adjusted *R*^2^**		**0.63**
High	Year of hybrid release	0.36	0.08	<0.001	8.00
	Factor 1	–0.31	−1.66	<0.001	8.00
	Factor 2	0.41	2.38	<0.001	12.0
	Factor 3	–0.08	−0.44	0.35	1.00
			**Adjusted *R*^2^**		**0.29**

*Partial correlations between predictor variables and per-area marketable ear mass (Mt/ha). Proportion of variance explained by each of the regression models is shown in bold.

## Discussion

Modern corn hybrids are plant-density dependent, i.e., yield gains are observed from using increased number of plants per unit area (Tokatlidis and Koutroubas, 2004). This is evident from increased optimal plant densities for modern field corn (Ciampitti and Vyn, 2012; Assefa et al., 2018) and certain crowding stress tolerant sweet corn hybrids (Dhaliwal and Williams, 2019). Our results using a sweet corn era panel show modern hybrids outperform old hybrids in per-area marketable ear mass at High plant density. These results are in agreement with previous findings utilizing field corn era panels, where yield gains were documented in modern hybrids at higher plant densities (Carlone and Russell, 1987; Duvick, 1997; Sangoi et al., 2002). Thus, gains in marketable ear mass observed in modern sweet hybrids are primarily due to increased PDT.

On the contrary, yield potential per plant has not changed in hybrid sweet corn since inception in the 1930s. Modern sweet corn hybrids did not show any yield superiority when plants were grown under conditions free of crowding stress. Similar results were reported from the analysis of field corn era hybrids under low plant densities (Duvick, 1997; Sangoi et al., 2002). Since evidence suggests that yield potential per plant has not changed in modern hybrids, growing modern hybrids at plant densities higher than their predecessors is essential to realize the benefits from improved PDT.

The era panel evaluated in this study comprised fresh-market, processing-type, and dual-purpose sweet corn hybrids; therefore, trends in yield metrics relevant to both fresh-market (crate yield) and processing industry (kernel mass and recovery) were quantified. Unlike crate yield, kernel mass showed limited improvement in modern hybrids. Recovery, the single most important variable to vegetable processors, showed no improvement over time at either density. Traditionally, sweet corn breeding programs have used ear number and mass to assess the performance of sweet corn hybrids; response variables unrelated to recovery (Williams, 2014). Recovery is vitally important to the vegetable processing industry, because as recovery increases, the processor buys less ear mass to achieve their “pack”—a seasonal goal of cases of finished product. Furthermore, efficiency of the processing factory improves with higher recovery (e.g., less husk and cob waste is generated). Hence, recovery should not be overlooked in evaluating processing sweet corn germplasm for improved PDT. Fortunately, in the last decade, measuring kernel mass and recovery has become more widespread to evaluate processing-type hybrids (M. Williams, pers. obs.; S. Grier, pers. com.).

Improved PDT is accompanied by changes in morphological traits that allow for use of more plants per unit area. Modern field corn hybrids have more compact plant architecture for reduced interference from neighboring plants at higher plant densities (Duvick, 2005; Ma et al., 2014). Our data show that modern sweet corn hybrids also tend to develop compact plant architecture under conditions free of crowding stress. For instance, modern sweet corn hybrids had fewer tillers per plant and lower fresh shoot biomass per plant. This modified plant architecture in modern sweet corn hybrids permits the utilization of more plants per unit area, and consequently higher LAI, and also ensures complete canopy closure.

Plant density tolerance is a complex trait in sweet corn. Choe et al. (2016) reported that the molecular basis of crowding stress tolerance in sweet corn is genotype specific, i.e., PDT hybrids have unique tolerance mechanisms. Gene expression studies identified a network of genes involved in biological functions including photosynthesis, glycolysis, cell wall structure, carbohydrate/nitrogen metabolic processes, chromatin, and transcription regulation-related processes as possible mechanisms of crowding stress tolerance in sweet corn. Our analysis of plant and ear traits showed that plant architecture—comprised of tillers per plant, LAI, and fresh shoot biomass per plant—predicted per-area marketable ear mass at Low density. Essentially, the more prolific sweet corn hybrids would yield higher per-area marketable ear mass under conditions free of crowding stress. However, morpho-physiological traits are poor predictors of PDT of specific hybrids, consistent with research on 26 modern *shrunken-2* hybrids (Williams, 2016). In short, modern hybrids with superior PDT cannot be identified from plant architecture alone.

Unlike field corn, morpho-physiological and ear traits in PDT sweet corn could not be structured into distinct categories like those previously identified by Mansfield and Mumm (2014). They classified 48 different plant morpho-physiological and ear traits into five categories: photosynthetic capability, plant architecture, growth responses, source–sink relationship, and general stress tolerance. The lack of explicit associations between underlying plant and ear traits, and PDT in sweet corn could be explained by inherently different breeding objectives for the two crops. Sweet corn breeders do not primarily select for yield, instead maintaining or improving eating quality and specific parameters for ear traits like ear length and girth and tip-fill. Sweet corn breeding also requires improving host plant resistance to common sweet corn diseases prevalent in the North America and focuses on post-harvest shelf life (Lertrat and Pulam, 2007; Pataky et al., 2011).

## Conclusion

To our knowledge, this is the first study to examine an era panel in sweet corn. We used the panel to quantify changes in PDT and associations with plant and ear traits. Our results show that modern sweet corn hybrids are plant density dependent, i.e., hybrids benefit from increased PDT under crowding stress. The increase in per-area marketable ear mass at the rate of 0.8 Mt/ha/decade in sweet corn is primarily due to improved PDT. Yield potential per plant has remained unchanged. Recovery has not changed over the last 80 years, likely because it was not the target of a breeding objective. Modern sweet corn hybrids have been modified into a generally more compact plant architecture that supports more individual plants per unit area and less interference from neighboring plants. However, plant architecture alone is not predictive of PDT among modern hybrids.

## Data Availability Statement

The original contributions presented in the study are included in the Supplementary Material, further inquiries can be directed to the corresponding author.

## Author Contributions

MW conceptualized and designed the study. DD led the overall study, contributed to the data collection, analysis, and interpretation, and wrote the manuscript. EA and MW contributed to the data collection and interpretation. All authors read, contributed to the manuscript revisions, and approved the final manuscript.

## Author Disclaimer

Any opinions, findings, conclusions, or recommendations expressed in this publication are those of the authors and do not necessarily reflect the view of the U.S. Department of Agriculture. Mention of trade names or commercial products in this publication is solely for the purpose of providing specific information and does not imply recommendation or endorsement by the U.S. Department of Agriculture. USDA is an equal opportunity provider and employer.

## Conflict of Interest

The authors declare that the research was conducted in the absence of any commercial or financial relationships that could be construed as a potential conflict of interest.

## Publisher’s Note

All claims expressed in this article are solely those of the authors and do not necessarily represent those of their affiliated organizations, or those of the publisher, the editors and the reviewers. Any product that may be evaluated in this article, or claim that may be made by its manufacturer, is not guaranteed or endorsed by the publisher.
