# Supplementary material for: Historical Trends in Sweet Corn Plant Density Tolerance Using Era Hybrids (1930–2010s)
Source: Front Plant Sci. 2021 Sep 22;12:707852. doi: 10.3389/fpls.2021.707852 (PMC8492913; doi:10.3389/fpls.2021.707852)

Supplementary Fig 1. Relative Humidity (%), daily water supply (mm), and average daily temperature (℃) for 2018, 2019, and 2020 growing seasons near Urbana, IL. Daily water supply is sum of daily precipitation and irrigation.

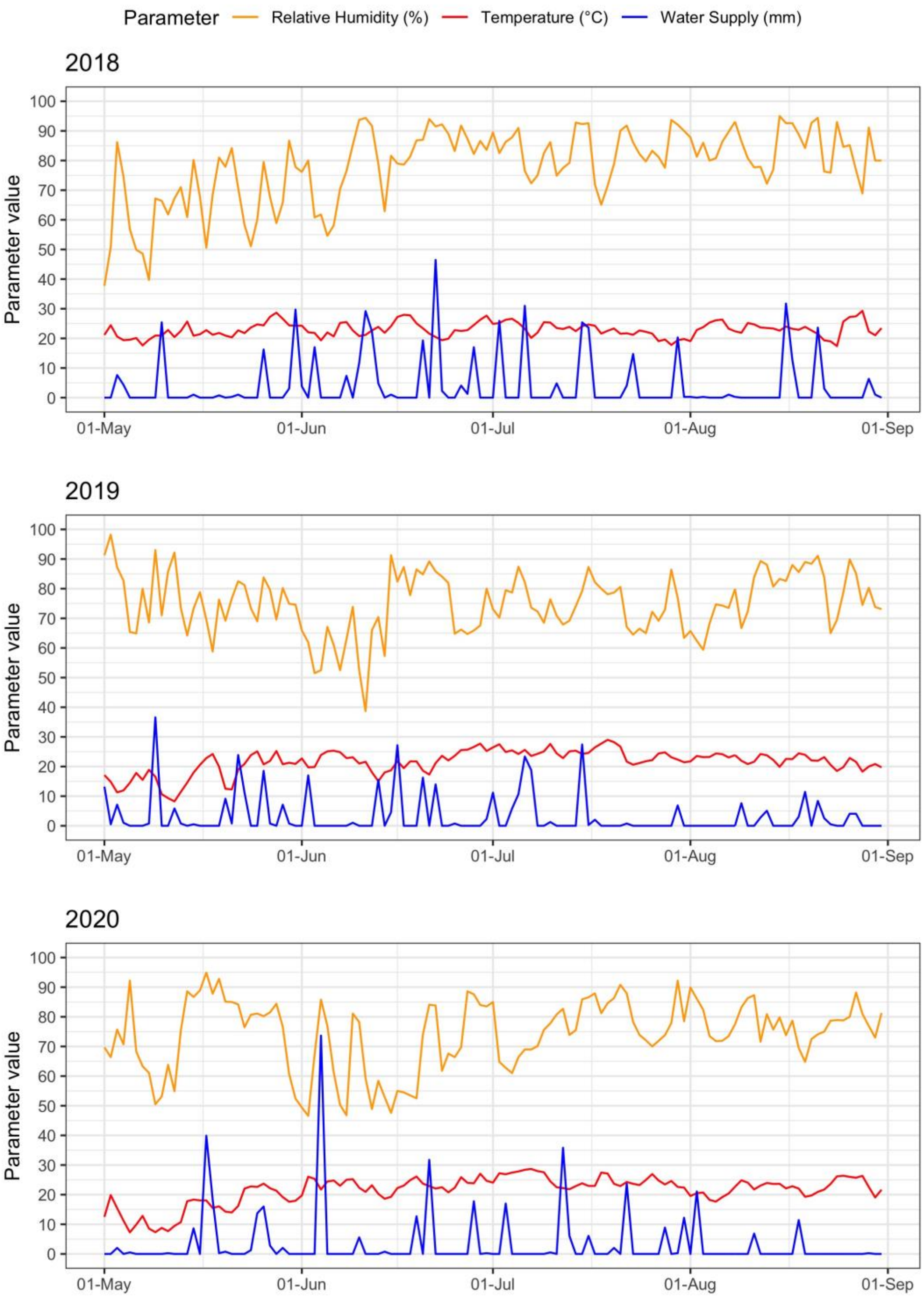

Supplement: Supplementary file 1 [file Data_Sheet_1.PDF]
